# The Impact of Social Cognition Deficits on Quality of Life in Multiple Sclerosis: A Scoping Review

**DOI:** 10.3390/brainsci14070691

**Published:** 2024-07-11

**Authors:** Giulia Marafioti, Davide Cardile, Laura Culicetto, Angelo Quartarone, Viviana Lo Buono

**Affiliations:** IRCCS Centro Neurolesi Bonino-Pulejo, S.S. 113 Via Palermo, C.da Casazza, 98124 Messina, Italyangelo.quartarone@irccsme.it (A.Q.); viviana.lobuono@irccsme.it (V.L.B.)

**Keywords:** multiple sclerosis, social cognition, quality of life

## Abstract

Background: Multiple Sclerosis (MS) is a chronic neurological disease that affects the Central Nervous System by causing demyelination. Social cognition (SC) deficits are common among individuals with MS and can significantly impact their quality of life (QoL) due to difficulties in interpreting social cues and establishing meaningful relationships. Objective: This scoping review aimed to investigate SC in subjects with MS and its impact on QoL. Methods: Systematic searches were performed in PubMed, Scopus, Embase, and Web of Science databases. After reading the full text of the selected studies and applying predefined inclusion criteria, four studies were included based on pertinence and relevance to the topic. Results: The findings highlight significant associations between SC deficits, social support, fatigue, and QoL outcomes. Cognitive decline was identified as a predictive factor for SC impairment in the MS population, which affects daily activities and relationships, thereby reducing QoL. Moreover, emotional impairments such as depression and anxiety exacerbate these challenges. Enhancing social support networks may improve psychological well-being and disease management in MS. Conclusions: Although evidence is limited, assessing SC is crucial in the care pathways for MS to develop tailored psychosocial interventions that address the cognitive, emotional, and social facets of the disease, thereby improving overall outcomes and QoL.

## 1. Introduction

Multiple Sclerosis (MS) is a chronic, degenerative neurological disease that is characterized by demyelination of the Central Nervous System (CNS) [1]. According to a report by the International MS Federation, the global median prevalence of MS rose from 30/100,000 in 2008 to 33/100,000 in 2013 [2]. This condition usually affects young adults in the age range of 20 to 40 years and is more prevalent in women than in men [3]. MS manifests in four main forms: relapsing-remitting (RRMS), secondary progressive (SPMS), primary progressive (PPMS), and relapsing-progressive (PRMS). RRMS is the most common form, affecting most MS subjects, and it is characterized by distinct relapses where new symptoms appear or existing ones worsen [4]. In contrast, PRMS lacks clear remission periods [5]. MS symptoms vary widely and are categorized as primary, secondary, and tertiary [5]. Primary symptoms directly result from demyelination and include visual problems, pain, spasticity, bowel/bladder issues, speech/swallowing difficulties, cognitive challenges, and fatigue. Secondary symptoms arise from complications of the primary symptoms and include difficulty with daily activities, balance issues, depression, and anxiety. Tertiary symptoms are due to the disease’s chronic nature, leading to social isolation, unemployment, and changes in roles and responsibilities.

Among the cognitive dysfunction in MS subjects, it has been reported to not only affect perceptual-motor functions, language, working memory, sustained attention, information processing speed, and executive functioning but also social cognition (SC) [6]. Social cognition encompasses the mental operations underlying social interactions, including the ability to perceive, interpret, and generate responses to the intentions, dispositions, and behaviors of others. This definition helps to distinguish it from broader cognitive functions such as memory, attention, and executive functions, which are commonly affected by cognitive impairment [7].

SC is a neurocognitive ability that includes different aspects of processing, decision-making, or response to the demands of social stimuli and can affect various neurological disorders [8,9]. Key components of SC include theory of mind (the ability to attribute mental states to oneself and others), emotion recognition (the ability to identify and respond to emotional expressions), empathy (the capacity to understand and share the feelings of another), and social perception (the ability to decode social cues and contexts). These cognitive processes are critical for effective communication and social functioning, and impairments in SC can significantly impact the quality of life (QoL) and social interactions of individuals with neurological disorders [10,11,12].

This multi-componential concept includes skills related to the Theory of Mind (ToM), empathy, and the social perception of emotions from prosody, facial expressions, and body gestures [13]. Difficulties in understanding the thoughts and feelings of others, known as ToM deficits, may be expected in cases of white matter alterations in the frontal and temporal cortical neural networks [14,15]. Prefrontal areas, indeed, contribute to the construction of an internal conscious representation of social situations through high-level processes such as ToM [16].

Social stimuli are also processed by other cortical and subcortical structures [17]. The inferior occipital cortex and inferior temporal regions are responsible for detecting the social valence of a stimulus and categorizing it based on its social properties [18]. The amygdala, hypothalamus, ventral striatum, and orbitofrontal cortex determine whether the stimulus is rewarding or punishing, as well as its emotional significance. Following this assessment, the individual can prepare the necessary autonomic and motor responses [18]. Frith and colleagues [19] showed how a neural network, consisting of the medial prefrontal cortex, the anterior paracingulate cortex, the superior temporal sulcus cortex (which extends into the parietal lobe), the temporo–parietal junction, and sometimes the temporal pole, is responsible for controlling cognitive and affective mentalizing abilities. Accumulating evidence suggests that subjects with MS are impaired in SC and presented alteration in non-verbal social skills and emotion recognition [20].

QoL is a broad, multidimensional concept that typically includes subjective evaluations of both positive and negative aspects of life. For individuals with chronic conditions such as MS, QoL encompasses physical health, psychological state, level of independence, social relationships, personal beliefs, and their relationship to significant features of their environment [21].

Several instruments can be used to assess QoL in people with MS, with the MusiQoL (Multiple Sclerosis International Quality of Life) and the SF-36 (36-Item Short Form Survey) being among the most widely used [22,23].

The impairment in SC has a significant impact on the QoL [24,25]. Some evidence has shown that the experience of typical symptoms of MS, coupled with dysregulation of SC and relationships, poses a significant challenge in adapting to everyday life, greatly affecting functional, physical, emotional, social, and spiritual well-being [26,27]. Despite this, the treatment and management of MS often focuses in a one-dimensional way: physical issues. Concentrating only on these aspects, however, does not allow due consideration to be given to psychological, cognitive, and social skill impairments and their effects on subjective well-being [28,29].

SC alterations are important predictors of decreased QoL in MS since they cause an impairment of the activities of daily living, and they are independent of motor disability. Given the significant impact that SC alterations can have on QoL in MS, this paper aims to evaluate whether and to what extent SC is assessed in studies involving subjects with this disease.

## 2. Methods

### 2.1. Search Strategy

**Figure 1:**
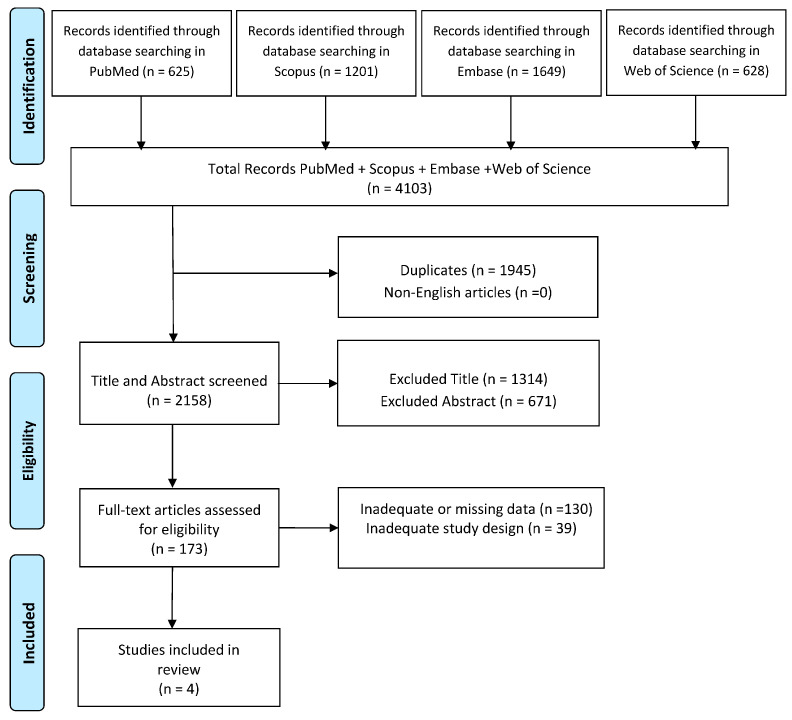
PRISMA diagram for the current review.

A scoping review of currently published studies was performed on 10 December 2023 using the following databases: PubMed, Scopus, Embase, and Web of Science. The search was carried out using the following search string: ((“social cognition” [All Fields] OR “cognition” [All Fields]) AND “multiple sclerosis” [All Fields] AND “quality of life” [All Fields]) AND ((humans [Filter]) AND (2000/1/1:2023/4/30 [pdat]) AND (English [Filter])). Initially, all articles were reviewed based on titles and abstracts by two investigators (G.M. and L.C.), who independently analyzed and collected data to reduce the risk of bias (e.g., language bias, publication bias, delay bias). Full-text articles deemed suitable for the study were then read by these researchers, and in cases of disagreements regarding inclusion or exclusion criteria, a final decision was made by a third researcher (V.L.B.). A PRISMA (Preferred Reporting Items for Systematic Reviews and Meta-Analyses) diagram was added to describe all steps of the search process (identification, screening, eligibility, and inclusion) for the collection and determination of qualified studies, as shown in Figure 1. Disagreements between reviewers were resolved by consensus.

### 2.2. Inclusion and Exclusion Criteria

An article was included if it involved adult subjects with MS. We selected only studies that investigated SC and QoL by standardized neuropsychological tests. Moreover, only articles written in English and published in a peer-reviewed journal were considered.

Animal studies, conference proceedings, and studies involving children were removed from this search. Case reports and systematic, scoping, or narrative reviews were also excluded.

## 3. Results

Electronic searches generated 1078 studies. Following the removal of duplicates, 566 studies were screened by title and abstract. Following full-text selection, four studies were included for analysis. The selection process is shown in Figure 1.

The studies selected have investigated SC and QoL in subjects with MS (Table 1). The standardized tests used to evaluate QoL and SC were Short Form Survey Version 2 (SF-36), International Affective Picture System (IAPS), Reading the Mind from the Eyes (RMET), Multiple Sclerosis Quality of Life 54 questionnaire (MSQOL54), Multiple Sclerosis International Quality of Life questionnaire (MusiQoL), Movie for the Assessment of Social Cognition (MASC), Faux Pas (FP), Emotion recognition (ER), Social Support Survey (MOS), Activities of Daily Living Questionnaire (ADLQ) (Table 2).

Eizaguirre et al. [30], in their study, examined a group of subjects consisting predominantly of women with different forms of MS. It was discovered that there are significant differences between those who have a better score in MOS and those with low scores. People with better scores tend to have more social support, while those with low scores tend to isolate themselves by failing to establish relationships and benefit from interpersonal relationships. Fatigue is also a significant factor that should not be underestimated, as tired subjects tend to experience greater physical and emotional instability, presenting obvious difficulties in everyday life. All these factors lead to psychological and social consequences. The authors found statistically significant differences in the size of the social network, where tired patients tended to have fewer friends than fatigued subjects. Additionally, the QoL is significantly different, resulting in decreased daily activities, relationships with friends and family, and relationships with love and sexual life. In another study [31], it was discovered that different levels of performance on ToM have an impact on people’s QoL. The cognitive rather than affective components of ToM influence QoL and the former are compromised in ToM. There was a significant correlation between ToM deficit and the subject’s cognitive performance, as demonstrated by neuropsychological evaluations. For those who have observed a decrease in ToM cognitive skills, these deficits are evident in both specific activities and real-life scenarios. In the study by Grothe et al. [32], individuals with acute disease relapse, psychiatric disorders, motor disabilities, and other CNS diseases were excluded. The authors focused only on subjects with MS, without a control group. The study found that cognitive functioning negatively impacted emotion recognition tasks and ToM. However, depression, fatigue, and disability were not related to SC. Additionally, SC did not appear to affect QoL. Crivelli et al. [33] examined 64 subjects divided into two groups: 30 healthy controls and 34 subjects with RRMS. The tests showed that SC was not affected by fatigue, neuropsychiatric disorders, or cognitive function, and there was no relationship between SC and QoL. The MS subjects had been experiencing the disease for only two years, yet SC impairment was observed even in these early stages. Significant differences between the two groups were found in cognitive tests, particularly in working memory and processing speed, while no differences were noted in tests assessing executive functions and empathy. Cognitive and social measures did not significantly differ in the results of ADLQ, but there was a positive correlation with anxiety measures.

## 4. Discussion

The purpose of this study is to examine the impact of SC alterations on QoL in people with MS. SC focuses on how people process, store, and apply information about others and social situations. It highlights the role that cognitive processes play in our social interactions. Understanding how we perceive and navigate social cues is fundamental to forming meaningful connections and managing chronic illnesses like MS. However, deficits in SC can disrupt these processes, leading to significant challenges in communication and social interaction. Research by Montel et al. [41] underscores the importance of SC in accurately interpreting social cues and establishing fulfilling social relationships, particularly in the context of managing chronic conditions. Moreover, some studies [42,43] emphasize the far-reaching consequences of SC deficits on QoL. These deficits not only hinder effective communication but also impede the formation of supportive social networks, exacerbating feelings of isolation and diminishing overall well-being. Critically examining SC within the framework of mental health sheds light on the intricate interplay between cognitive processes and social functioning. By addressing these deficits, interventions can potentially enhance individuals’ QoL by fostering better social adaptation and bolstering coping mechanisms.

Although the construct is of great interest, few literature studies have investigated the impact that alterations in SC can have on the QoL in people with MS.

This review underscores the notable disparities in QoL among individuals with MS, manifesting in disruptions across various facets of daily life. These disruptions extend to challenges in maintaining interpersonal relationships with family and friends, engaging in sentimental connections, and addressing sexual aspects of life [30,32], contributing to this understanding by highlighting substantial differences in ToM and emotion recognition abilities between individuals with MS and the healthy population. These cognitive capacities are pivotal for comprehending and appropriately responding to the emotions and mental states of others, essential components for fostering effective social interactions. This discrepancy in ToM and emotion recognition abilities further underscores the intricate relationship between SC deficits and the challenges experienced by individuals with MS in navigating social interactions and preserving QoL [33].

People with MS consistently performed worse on tasks measuring ToM and emotion recognition compared to healthy controls [44]. Importantly, these performance deficits could not be explained by general cognitive decline, neuropsychiatric symptoms such as anxiety and depression, or fatigue, which are common concerns in MS subjects. This indicates that the observed differences are likely due to specific impairments in SC directly associated with MS [20,45]. Furthermore, this study uncovered a significant link between lower scores on tests involving the interpretation of unusual emotional stories and variations in both physical and mental QoL levels among individuals with MS. This finding underscores the crucial impact of social and mental capabilities on the overall well-being of MS subjects. Essentially, impairments in understanding and processing emotional and social information can lead to diminished QoL, highlighting the importance of addressing these social cognitive deficits in therapeutic interventions aimed at improving the lives of those with MS [33].

The authors from different studies suggest that cognitive decline impacts both SC and QoL [31,32]. Among the resulting data, findings corroborate earlier research, indicating that cognitive impairment is a predictive factor for the decline of ToM in MS individuals [9,46]. Managing fatigue is crucial for people with MS, as it can cause physical and emotional instability, reduce QoL, and lead to social isolation.

Researchers are increasingly investigating the repercussions of SC impairment on daily life within chronic neurodegenerative conditions [45].

While some studies have explored the potential connection between QoL and SC, much of the research remains generalized and imprecise. Therefore, there is a pressing need to emphasize evaluating SC, particularly in the early stages of MS, to better understand its impact on QoL and develop tailored psychosocial interventions to improve patient outcomes. However, it is very difficult to isolate one element (such as social cognition) and estimate what impact it has on the quality of life of patients with multiple sclerosis. This difficulty is due to the fact that there are many elements that are present in the disease, and they are closely related.

Understanding how SC alterations affect QoL is essential for advancing the development of effective psychosocial interventions. In addition to cognitive and social challenges, MS subjects also face specific difficulties related to emotional regulation. Emotional disturbances, including depression and anxiety, are prevalent among individuals with MS and can significantly impact their overall well-being and QoL [47]. Emotional symptoms are often intertwined with the physical and cognitive manifestations of the disease, creating a complex landscape that requires comprehensive treatment approaches. Furthermore, emerging research suggests a bidirectional relationship between social support and QoL in MS subjects.

Adequate social support has been associated with better QoL outcomes, including improved psychological well-being and disease management [48]. Therefore, interventions aimed at enhancing social support networks may play a crucial role in optimizing QoL for individuals living with MS. In summary, MS poses multifaceted challenges beyond physical symptoms, encompassing cognitive, emotional, and social dimensions. Recognizing and addressing these diverse aspects of the disease are essential for improving overall outcomes and enhancing the QoL of individuals affected by MS.

Among the instruments used to measure QoL are the MusiQoL and the SF-36, but they have some limitations that should be acknowledged. The MusiQoL, despite being designed for international use, can still be affected by cultural differences that influence how individuals respond to the questions, potentially impacting the comparability of results across different populations. Additionally, while MusiQoL covers various aspects of QoL, it may not fully capture all dimensions of the disease’s impact, such as cognitive functions or specific emotional challenges faced by MS patients. Furthermore, the fixed structure of the MusiQoL might not be flexible enough to adapt to the evolving nature of MS and the diverse experiences of patients over time. Similarly, the SF-36, a generic QoL instrument, is designed for the general population and various diseases, not specifically for MS. This limits its sensitivity to the specific issues and changes in QoL that are particularly relevant to the MS population. Additionally, the SF-36 emphasizes physical functioning, which may overshadow other critical aspects of MS, such as mental health, cognitive function, and social interactions. Furthermore, it may not be sensitive enough to detect small but clinically meaningful changes in QoL over time in MS patients, given the chronic and fluctuating nature of the disease.

This review shows that although SC has been studied extensively, evidence of its evaluation in care pathways is still scarce. It is important to note that while various articles discuss SC, there is often significant confusion between the terms SC and social support. Many times, studies intended to examine SC end up focusing on social support instead, leading to a conflation of these distinct concepts. This confusion underscores the need for clearer definitions and more precise research methodologies in future studies.

Additionally, understanding the distinction between these terms is crucial for developing targeted interventions. SC refers to the processes involved in understanding and interacting with others, which include empathy, theory of mind, and social perception. In contrast, social support pertains to the actual and perceived availability of resources and assistance from others. Clarifying these differences can help in designing more effective strategies that address both the cognitive and supportive needs of individuals with MS.

Our study results highlight the complexity of discerning between SC and general cognitive functioning. This difficulty arises because both constructs intertwine within a bio–psycho–social framework. The cognitive functions involved in SC include attention, memory, executive functions, perception and understanding of emotions, and theory of mind. Therefore, a proper evaluation of SC warrants a thorough assessment of these related areas. Consequently, a sensitive and standardized battery should holistically consider all these measures.

Among the limitations of this review are perhaps the overly stringent inclusion criteria and the resulting small number of studies included. However, the latter, in our opinion, is also a strength. The small pool of studies in fact confirms that there is still no standardized protocol in the literature that considers the many variables that should be given due consideration in the management of a complex patient such as one with MS. In the future, we aim to extend the list of databases to be taken into consideration so that we can possibly compare several measures to be able to decide which one is more sensitive or to lay the foundations for a new standardized instrument that is inclusive of all aspects. The strong impact that SC has on MS subjects and on their QoL is often amplified by co-occurrent psychological disturbances like depression and anxiety. Deepening research on these issues means moving toward the discovery of increasingly inclusive treatment approaches. Enhancing social support networks can lead to better psychological well-being and disease management among individuals living with MS. There is a pressing need for more focused research and tailored psychosocial interventions aimed at addressing cognitive, emotional, and social aspects of MS to enhance overall outcomes and QoL. Treating individuals holistically allows for personalized management of the people’s health status according to a bio–psycho–social approach.

## Figures and Tables

**Table 1:** Main results of the selected studies.

Authors	Aim	Methods/Treatments	Instruments	Subjects	Outcomes
Eizaguirre, M. B., et al. (2020) [30]	Examine the correlation between perceived fatigue, social support, cognition, and QOL in an Argentine population of MS subjects.	Neuropsychological evaluation examining the relationship between perceived fatigue and QoL, social support, and cognition.	- FSS - EDSS - MusiQoL - CVLT - BVMTR - PASAT-3 - MOS	128 (75 females), age: 40 ± 1049	The objective was to study the relationship between perceived fatigue and QoL, social support, and cognition in an Argentine population of patients with MS.
Isernia, S, et al. (2019) [31]	Investigate the relationship between ToM, clinical variables, and neuropsychological profile in a cohort of adults with long-standing diseases.	Screening with a neuropsychological and ToM battery, evaluating both the affective and emotional cognitive components.	- EDSS - RMET - MoCA - BRB-NT - SRT-LTS - SRT-CLTR - SPART - SDMT - PASAT 2/PASAT 3 - SRT-D - SPART-D - BDI-II - STAI-Y1 - PARADISE 24 - MSQOL54 - SF-36 - MASC - SS	42 (24 females) age: 52.38; 26 HC (19 females) age: 51.35	The aim of this study was to evaluate the relationship between ToM, clinical variables (duration of the disease and level of disability), and neuropsychological profile.
Grothe, M, et al. (2021) [32]	The aim of this study was to compare two SC tests among patients with MS and other clinical variables.	Two tests assessing SC, emotion recognition, and ToM were administered. QoL assessment was also conducted.	- EDSS - BDI - MusiQoL - BRB - SDMT - ER	50 (29 females), age: 39.4 ± 9.7	This study compared two tests of SC in people with MS with respect to other clinical variables. The impact that SC has on patients’ QoL was also investigated.
Crivelli L, et al. (2024) [33]	To investigate the influence of cognitive performance, fatigue, and neuropsychiatric symptoms on SC performance in early MS patients with low EDSS and to assess their QoL.	Neuropsychological assessment that included tests of SC.	- MusiQoL - ADLQ - PASAT - RMET - IAPS - FP - EQ	64 patients, of which: 34 patients with relapsing-remitting MS, with disease duration 2 years and scores of 2 at EDSS; 30 healthy controls. age: 34, 34.71 (8.17)	This study assessed the impact of MS on SC and QoL.

Legend: HC: healthy controls; Fatigue Severity Scale (FSS); Expanded Disability Status Scale (EDSS); Sclerosis International Quality of Life questionnaire (MusiQoL); California Verbal Learning Test (CVLT); Brief Visuospatial Memory Test—Revised (BVMTR); Rao adapted 3-s (PASAT-3); Social Support Survey Multiple (MOS); Reading the Mind from the Eyes (RMET); Montreal Cognitive Assessment (MoCA); Repeatable Battery of Neuropsychological Test (BRB-NT); Selecting Reminding Test—Long Term Storage (SRT-LTS); Reminding Test—Consistent Long Term Retrieval (SRT-CLTR); Spatial Recall Test (SPART); Symbol Digit Modalities Test (SDMT); Paced Auditory Serial Addition Test (PASAT 2/PASAT 3); Selective Reminding Test (SRT-D); Spatial Recall Test (SPART-D); Beck Depression Inventory-II (BDI-II); The Brief Repeatable Battery of Neuropsychological Tests (BRB; Paced Auditory Serial Addition Test (PASAT); State-Trait Anxiety Inventory—Y1 (STAI-Y1); PARADISE 24; Multiple Sclerosis Quality of Life 54 questionnaire (MSQOL54); Short Form Survey version 2 (SF-36); Movie for the Assessment of Social Cognition (MASC); Strange Stories (SS); Symbol Digit Modalities Test (SDMT); Emotion recognition (ER); Activities of Daily Living Questionnaire (ADLQ); International Affective Picture System (IAPS); Faux Pas (FP); Empathy Quotient (EQ); Theory of Mind (ToM); quality of life (QoL).

**Table 2:** Neuropsychological assessment of social cognition and quality of life.

Subjective Tools	Description/Structure
Short Form 36 (SF-36) [23]	A self-administered questionnaire that quantifies the individual’s health status and assesses the health-related QoL. It comprises 36 items and two summary scores: physical and mental.
International Affective Picture System (IAPS) [34]	A comprehensive collection of emotionally evocative color photographs. These images are designed to evoke a range of emotional responses and are accompanied by assessments of pleasure, excitement, and dominance, as expressed by both men and women.
Reading the Mind from the Eyes (RMET) [11,26]	A task involving 36 visual stimuli, typically photographs depicting the eyes of actors expressing various basic or complex emotions. The total score on this task ranges from 0 to 36, reflecting the accuracy of participants’ recognition of emotions based on the depicted eye expressions.
Multiple Sclerosis Quality of Life 54 Questionnaire (MSQOL-54) [35]	A multidimensional health-related QoL instrument specific to MS. It is composed of 52 items distributed in 12 scales.
Multiple Sclerosis International Quality of Life Questionnaire (MusiQoL) [36]	A self-administered tool designed to assess the QoL in patients with MS. It consists of 31 items, organized into nine dimensions: activities of daily living, psychological well-being, symptoms, friendship relationships, family relationships, satisfaction with health care, love and sexual life, coping, and denial.
Movie for the Assessment of Social Cognition (MASC) [37]	A 15 min film program depicting four characters gathering for a dinner party. Throughout the film, the video is paused 46 times, and subjects are prompted with questions regarding the characters’ feelings, thoughts, and intentions.
Faux Pas (FP) [37]	A task that entails listening to a series of stories comprising 4 faux pas narratives and 4 control stories. Following each story, participants are tasked with answering 6 questions designed to assess various aspects of their social cognition abilities. Each story is scored out of 6, resulting in a maximum total score of 24 across all stories. This scoring system allows for a comprehensive assessment of the participant’s ability to understand social cues, interpret intentions, and empathize with others in various narrative contexts.
Ekman 60-Faces Test [38]	A task of facial expression identification using the Ekman and Friesen Stimulus Set, which is a standardized set of black and white photographs. The set includes pictures of actors posing the six fundamental emotions of happiness, surprise, fear, sadness, disgust, and anger, as well as neutral facial expressions.
Social Support Survey (MOS) [39]	A self-administered multidimensional scale used to assess social support. It encompasses four distinct categories of social support: emotional/informational support, tangible support, positive social interactions, and affective support.
Activities of Daily Living Questionnaire (ADL) [40]	A questionnaire that provides a complete assessment of the patient’s functional abilities in various areas of daily life. It is structured into six sections. Within each section, there are three to six elements, which represent different tasks or behaviors relevant to daily functioning. Participants rate each item on a 4-point scale, ranging from 0 (indicating no problem performing the activity) to 3 (indicating inability to perform the activity). The total score ranges from 0 to 100.
